# Assessing soil properties and nutrient availability under conservation agriculture practices in a reclaimed sodic soil in cereal-based systems of North-West India

**DOI:** 10.1080/03650340.2017.1359415

**Published:** 2017-08-23

**Authors:** H. S. Jat, Ashim Datta, P. C. Sharma, Virender Kumar, A. K. Yadav, Madhu Choudhary, Vishu Choudhary, M. K. Gathala, D. K. Sharma, M. L. Jat, N. P. S. Yaduvanshi, Gurbachan Singh, A. McDonald

**Affiliations:** a Sustainable Intensification Programme, International Maize and Wheat Improvement Center (CIMMYT), New Delhi - 110012, India; b Division of Soil and Crop Management, ICAR-Central Soil Salinity Research Institute (CSSRI), Karnal - 132001, Haryana, India; c Division of Crop Improvement, ICAR-Central Soil Salinity Research Institute (CSSRI), Karnal - 132001, Haryana, India; d Sustainable Intensification Programme, International Maize and Wheat Improvement Center (CIMMYT), Dhaka, Bangladesh; e Division of Soil Science and Agricultural Chemistry, Indian Agricultural Research Institute (IARI), Pusa, New Delhi - 110012, India; f Ex-Chairman, Agricultural Scientists Recruitment Board (ASRB), Pusa, New Delhi - 110012, India; g Sustainable Intensification Programme, International Maize and Wheat Improvement Center (CIMMYT), Kathmandu, Nepal

**Keywords:** Cereal based systems, conservation agriculture, soil properties, available macro and micro nutrients, nutrient omission study

## Abstract

Soil quality degradation associated with resources scarcity is the major concern for the sustainability of conventional rice-wheat system in South Asia. Replacement of conventional management practices with conservation agriculture (CA) is required to improve soil quality. A field experiment was conducted to assess the effect of CA on soil physical (bulk density, penetration resistance, infiltration) and chemical (N, P, K, S, micronutrients) properties after 4 years in North-West India. There were four scenarios (Sc) namely conventional rice-wheat cropping system (Sc1); partial CA-based rice-wheat-mungbean system (RWMS) (Sc2); CA-based RWMS (Sc3); and CA-based maize-wheat-mungbean (Sc4) system. Sc2 (1.52 Mg m^−3^) showed significantly lower soil bulk density (BD). In Sc3 and Sc4, soil penetration resistance (SPR) was reduced and infiltration was improved compared to Sc1. Soil organic C was significantly higher in Sc4 than Sc1. Available N was 33% and 68% higher at 0–15 cm depth in Sc3 and Sc4, respectively, than Sc1. DTPA extractable Zn and Mn were significantly higher under Sc3 and Sc4 compared to Sc1. Omission study showed 30% saving in N and 50% in K in wheat after four years. Therefore, CA improved soil properties and nutrient availability and have potential to reduce external fertilizer inputs in long run.

## Introduction

Rice-wheat is a major cropping sequence in the Indo-Gangetic Plains (IGP) of South Asia; covering over 13.5 million ha in Bangladesh, India, Nepal and Pakistan and source of livelihood to millions of people (Ladha et al. 2000; Timsina and Connor 2001). The problems of post Green Revolution due to intensive farming, imbalance use of fertilizers and faulty irrigation practices cause soil degradation and depletion of soil organic carbon (SOC), water resources and environment pollution leading to stagnation or decline in yields of the rice-wheat cropping system (RWCS) in many parts of South Asia (Timsina and Connor 2001; Ladha et al. 2003). Cultivation of conventional puddled rice has led to over-exploitation of groundwater leading to an alarming fall of water table in many parts of North-western India (Humphreys et al. 2010). This necessitates for immediate solution through adoption of best management practices for improving soil and environment quality, and maintaining ecosystem services. As an alternative to conventional practices, CA have shown its effectiveness in sustaining and improving productivity of RWCS at the same time preserving scarce natural resources such as energy, labour, time, water and environment quality (Dikgwatlhe et al. 2014). Thierfelder and Wall (2009) showed the efficiency of CA systems in slowing down the soil physical, chemical and biological quality degradation while reducing cost of production.

Sustaining productivity of RWCS cannot be maintained unless the declining trend in soil fertility resulting from the nutrient mining by these crops is replenished (Subehia and Sepehya 2012). Increasing fertilizer costs as well as shrinking sources of organic manures (e.g. farm yard manure) along with inadequate input availability caused suboptimal nourishment of agricultural soils. In IGP of North-west (NW) India nearly 44.5 Mt rice residues and 24.5 Mt of wheat straw are burned annually (Singh and Sidhu 2014). Burning of crop residues is a serious concern in NW India as major N, S and C fractions in the residue are lost during burning and it accelerates the losses of organic matter, increases CO_2_ emissions, and reduces soil microbial activity (Biederbeck et al. 1980). Crop residues retention at soil surface conserves soil and water for sustaining crop production (Wilhelm et al. 2007), and increases SOC, thereby improving soil properties such as soil structures, cation exchange capacity, water holding capacity and lower bulk density. In this aspect, CA could be a better alternative which not only utilizes crop residues at the same time recycles plant nutrients in soil, improves soil properties and provides environmental benefits by avoiding in-situ burning.

Furthermore, the conventional puddled transplanted rice (PTR) requires large amount of energy and labour (Bhushan et al. 2007) as well as consumes larger quantities of irrigation water, and affects physical and chemical soil properties thereby adversely influencing productivity of the succeeding upland crop (e.g. wheat) (Gathala et al. 2011). This calls for development of a crop production technology throughout Asia to explore rice production technologies that will avoid puddling, require less water, save labour for transplanting, maintain rice yield potential and are environmentally friendly. Direct dry seeding of rice (DSR) into soil has proved to be an appropriate alternative to manually transplanted PTR (Prasad 2011). Maize due to its higher water use efficiency can be an excellent alternative to PTR in NW India where lowering of ground water level is a grave concern. Mungbean grown in between wheat and rice is beneficial in enhancing the carbon and nitrogen concentration in soil thereby improving the overall soil quality (Singh et al. 2015).

Information on soil properties, especially physical properties and nutrient availability under different agricultural management systems is essential for sustainability of the systems (Singh et al. 2014). There is an improvement in numerous parameters of soil health (physical, chemical and biological quality) under conservation tillage by increasing carbon and nutrients concentration at the surface soil (Singh et al. 2015; Salahin et al. 2017). Advantages of zero tillage (ZT) after burning or removal of crop residues in the RWCS were reported in the IGP, particularly in NW India (Erenstein and Laxmi 2008). In most of the cases ZT is practised in wheat for the timely sowing of wheat, control of *Phalaris minor*, reducing cost of cultivation and water saving without taking into account improvements in soil properties and nutrient availability. Studies on changes in macro and micro nutrient availability as well as soil properties under different CA-based practices in RWCS are very limited. Information on changes in soil properties and nutrient availability as well as nutrient savings through nutrient omission experiment under different CA-based practices in IGP is also limited. Therefore, an attempt was made to study the effect of CA practices on selected soil properties and nutrient availability and nutrient (N and K) savings after 4 years of cereal-based cropping systems in western IGP of India.

## Materials and methods

### Study site characteristics

**Figure 1.**
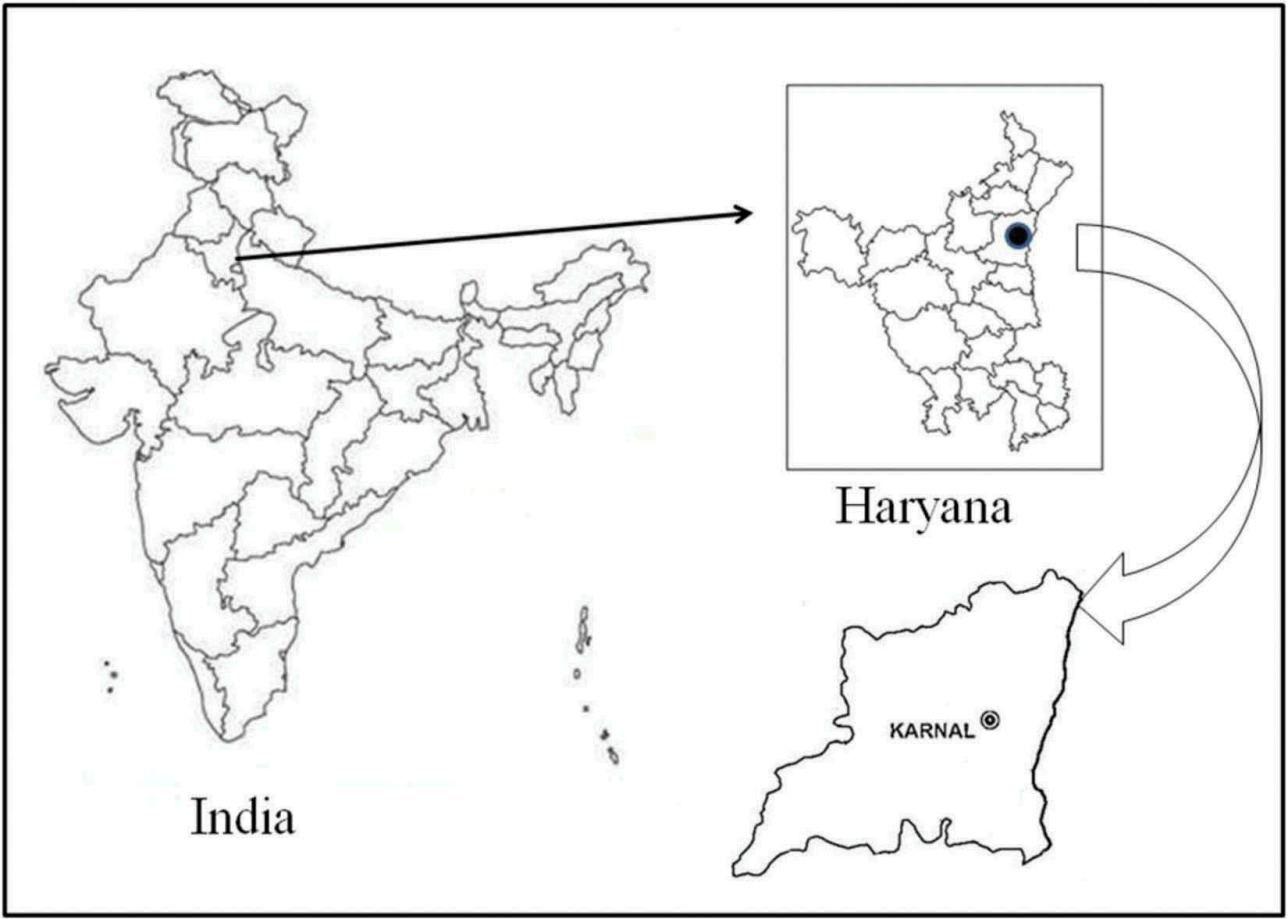
Location of the experiment.

A field experiment was conducted during 2009–2013 on the CIMMYT (International Maize and Wheat Improvement Centre) – CSSRI (Central Soil Salinity Research Institute) strategic research platform – located at CSSRI (29° 42ʺ20.7ʹ N latitude, 76° 57ʺ19.79ʹ E longitude and at an elevation of 243 m above msl), Karnal, Haryana, India (Figure 1). Climate of the region is semi-arid and sub-tropical with extreme hot and dry (April-June) to wet summers (July-September) during the mungbean (*Vigna radiata* L.) and rice (*Oryza sativa* L.) /maize (*Zea mays* L.) growing periods and cold dry winters (October-March) for wheat (*Triticum aestivum* L.), with an average annual rainfall of 670 mm, 75–80% of which is received during monsoon season. The soil is Haplic Solonetz (siltic) (WRB 2006). It was highly sodic with high pH (1:2.5) of 10.3 and exchangeable sodium of 97% at 0–5 cm depth in 1970. ICAR-Central Soil Salinity Research Institute was established in 1969 and developed reclamation technologies particularly addition of gypsum and adoption of best management practices for cereals, thereby the soils were reclaimed and cultivation of crops started (Datta et al. 2015). The soil of the experimental field was loam in texture, low in organic carbon with slightly alkaline pH. The initial soil characteristics of the experimental site can be obtained from Gathala et al. (2013).

### Treatments and experimental design

The treatments included four cereal-based cropping systems/scenarios varying in cropping system, tillage, crop establishment methods, water, nutrient and crop residue management practices. Treatments were organized in a randomized complete block design and replicated thrice in production-scale plots, each measuring 2000 m^2^ (20 m × 100 m). The scenarios (Sc) were designed keeping in view present as well as future drivers of agricultural changes in the region and their details can be obtained from Gathala et al. (2013).

In Sc1 (conventional) both rice and wheat were established with conventional practice, rice by manual transplanting of 30-day-old seedlings in puddled soil and wheat by manual broadcasting in tilled and pulverized soil. Scenario2 (partial CA-based rice-wheat-mungbean system) consisted of conventional practice of transplanting in puddled soil and subsequent wheat and mungbean by drill seeding in zero-till conditions. Under Sc3 (CA-based rice-wheat-mungbean system) and Sc4 (CA-based maize-wheat-mungbean system) all the three crops were drill seeded in ZT conditions. Details relating to cropping system, tillage, residue retention, crop establishment, variety, irrigation and fertilizer management practices are described in Gathala et al. (2013). Best bet (recently developed recommended practices for nutrient, water, weed, pests etc.) crop management practices were followed in all the scenarios except Sc1, where farmer’s practices were followed. Weeds were managed using herbicides in rice, maize and wheat crop in the initial years but after 3 years no herbicides applied in wheat under Sc3 and Sc4 because the weed population was negligible.

### Soil sampling and processing

Soil samples were collected from 0–15 and 15–30 cm soil depths using auger with 5 cm internal diameter after harvesting of rice in 2013 after four years of the experiment. Each plot was divided into four of 50 m × 10 m grid. Within each grid, a composite sample of each depth was prepared by taking samples from nine locations. The soil samples were air-dried in shade, ground to pass through a 2-mm sieve, stored in plastic jar for laboratory analysis of selected soil chemical properties.

### Measurement of soil parameters

Soil textural analysis was performed by following International Pipette Method (Baruah and Barthakur 1999). The textural class was determined by the United States Department of Agriculture (USDA) system (Soil Survey Division Staff 1993). Soil bulk density was measured by core method using 5 cm long and 5 cm internal diameter metal cores by placing the core in the middle of each soil layer (Blake and Hartge 1986) after harvest of rice in October 2013. Soil moisture was determined by gravimetric method and volumetric water content was calculated by multiplying gravimetric water content with bulk density of each layer (Baruah and Barthakur 1999). Soil penetration resistance was determined to a depth of 45 cm at every 5 cm depth interval using a manual cone penetrometer (Eijkelkamp Agrisearch Equipment, Germany). The cumulative infiltration and infiltration rate were determined by double-ring infiltrometer as described by Gathala et al. (2011).

Soil pH and electrical conductivity (EC) in soil: water ratio of 1:2 were determined by following standard methods (Jackson 1973). The organic carbon (OC) content of the soils was determined using wet oxidation method (Walkely and Black 1934), and total nitrogen (N) concentration was estimated by CHNS Vario El III analyser (Elementar, Germany). The available N in soil was determined by alkaline permanganate method (Subbiah and Asija 1956), available phosphorus (P) by ascorbic acid reductant method of Olsen et al. (1954), available potassium (K) by flame photometer using neutral 1*N* ammonium acetate extractant as described by Jackson (1973), available sulphur (S) by turbidimetric barium chloride procedure as described by Chesnin and Yien (1951). Available (DTPA-extractable) Fe, Mn, Zn and Cu of the soil samples were estimated by using atomic absorption spectrophotometer (AAS) following the method of Lindsay and Norvell (1978).

### Nutrient omission study

Within the scenarios, nutrient omission trial was conducted to study the effect of improved soil physical and chemical properties on response (yield) of wheat to varying levels of N and K fertilization after four years of continuous cultivation. A block of 2 m × 15 m size was selected in the main production scale plot of 2000 m^2^ and replicated as main scenario. The treatments comprised of different rates of N (100%, 85%, 70%, 55% and 0%) and K (100%, 50% and 0%) of the recommended dose of 160 and 60 kg N and K ha^−1^, respectively. Sources of N and K were urea (46% N) and muriate of potash (KCl) (60% K). At wheat maturity, complete selected block (30 m^2^) for nutrient (N and K) omission study were harvested manually. Grain yield of wheat was recorded at 14% moisture content basis.

### Statistical analysis

Data were subjected to analysis of variance (ANOVA) and analyzed using the SPSS window version 17.0 (SPSS Inc., Chicago, USA). Treatment means were separated by Duncan Multiple Range Test at 5% level of significance. A Pearson’s correlation matrix was constructed among the soil properties studied.

## Results and discussion

### Volumetric water content

CA based scenarios significantly influenced soil water content under Sc2, Sc3 and Sc4 in comparison to Sc1 in both the soil depths (Table 1). The Sc2, Sc3 and Sc4 (ZT with surface residue retention) had higher soil water contents, especially in the upper 0–15 cm depth. Volumetric water content at 0–15 cm soil depth was increased (P = 0.05) by 3.7% under Sc2, 8.9% under Sc3 and 7% under Sc4 than Sc1. This increase in water content in surface soil depth might be due to the mulching effect of crop residues, thereby reducing evaporation and conserving soil moisture. A lower volume of macropores and a higher volume of medium sized pores are also the possible reasons for higher soil water content under CA-based scenarios compared to conventional practice (Malecka et al. 2012). Water content at lower soil depth increased significantly by 45.2% under Sc2 as compared to Sc1. The greater increase in soil water content at lower depth of Sc2 may be attributed to the puddling in rice and crop residue incorporation, which led to the formation of impervious layer restricting water movement below 30 cm soil depth. Higher volumetric water content in surface soil under different CA-based practices was also reported by other researchers (Malecka et al. 2012; Wahbi et al. 2014).

**Table 1.** Volumetric water content, soil bulk density, pH, EC and organic C in different soil depths as affected by different CA based scenarios after harvesting of rice, 2013.

	Volumetric water content (%)		Bulk density (Mg m^−3^)		pH		Electrical Conductivity (dS m^−1^)		Oxidizable Organic C (g kg^−1^)	
Scenarios^a^	Soil depth (cm)									
	0–15	15–30	0–15	15–30	0–15	15–30	0–15	15–30	0–15	15–30
1	17.7 ± 1.7c	25.2 ± 9.0b	1.67 ± 0.01a	1.78 ± 0.06a	8.06 ± 0.06a	8.36 ± 0.21a	0.21 ± 0.04a	0.22 ± 0.07ab	4.5 ± 0.02b	3.5 ± 0.01b
2	21.4 ± 1.9b	36.6 ± 3.3a	1.52 ± 0.03b	1.61 ± 0.05b	7.70 ± 0.26b	8.22 ± 0.13a	0.20 ± 0.06a	0.33 ± 0.02a	5.4 ± 0.02b	4.9 ± 0.18a
3	26.6 ± 1.6a	28.6 ± 1.4ab	1.63 ± 0.02ab	1.80 ± 0.02a	7.84 ± 0.16ab	8.36 ± 0.24a	0.25 ± 0.05a	0.24 ± 0.12ab	7.5 ± 0.15a	3.6 ± 0.05b
4	24.7 ± 2.0ab	27.6 ± 1.5ab	1.64 ± 0.13ab	1.76 ± 0.01a	7.60 ± 0.12b	8.27 ± 0.10a	0.26 ± 0.03a	0.19 ± 0.03b	7.7 ± 0.08a	3.6 ± 0.16b

^a^Sc 1- conventional rice-wheat system, Sc 2- partial CA-based rice-wheat-mungbean system, Sc 3- CA-based rice-wheat-mungbean system, Sc 4- CA-based maize-wheat-mungbean system. For all variables n = 3 ± standard deviation. Values within the same column differ significantly at P = 0.05 when not followed by the same small letter (s) according to Duncan Multiple Range Test for separation of mean.

### Bulk density

Soil BD was significantly lower in Sc2 (1.52 and 1.62 Mg m^−3^ at 0–15 and 15–30 cm, respectively) compared to other scenarios at both the soil depths (Table 1). Differences in soil BD were not significant among Sc1, Sc3 and Sc4 in both the soil depths. Non-significant decrease in bulk density under Sc3 and Sc4 than Sc1 may be due to the age of the experiment (4 years) which might not be sufficient to reduce the BD significantly (Table 1). In CA based scenarios there was almost no disturbance in soil as well as all the agricultural operations were done mechanically. Under this situation residues retained on soil surface did not significantly reduce BD. Similar result for soil bulk density was reported by McVay et al. (2006) who found that below 3 cm soil depth BD values were only minimally affected by management practices. At lower depths under conventional tillage, higher BD might be due to the formation of a traffic pan. Higher BD at surface soil of Sc3 and Sc4 than Sc2 might be due to machine induced compaction (McVay et al. 2006). Soils higher in SOC are not prone to compaction, which may help explain why so little change in bulk density occurred at the CA-based scenarios (McVay et al. 2006). The highest decrease in BD in soil depths under Sc2 in comparison to Sc1 is attributed to increase in soil organic matter content, increase in activities of soil micro-and macro-organisms and improved aggregation due to well mixing of residues with soil particles (Singh and Sidhu 2014).

### Soil penetration resistance

**Figure 2.**
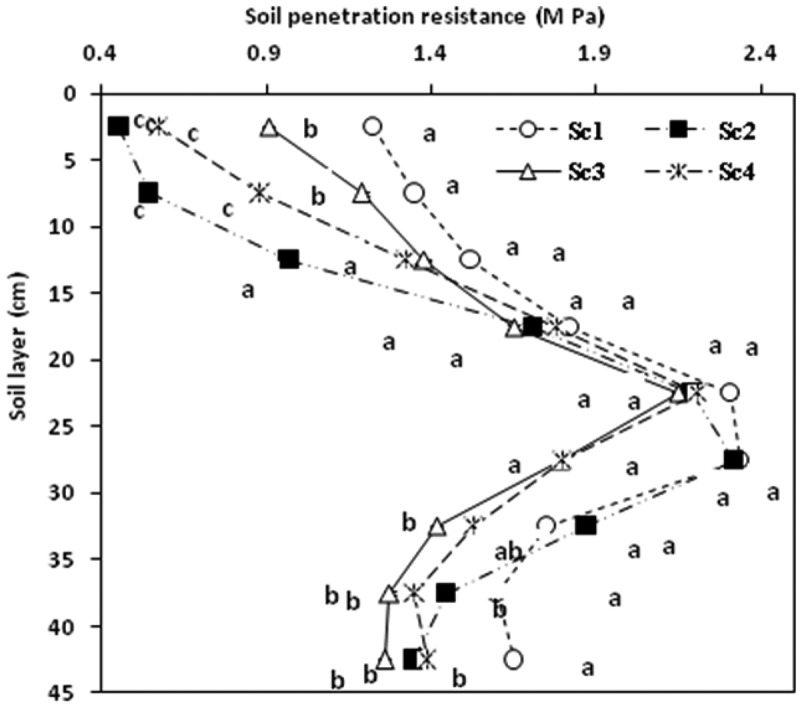
Soil penetration resistance (MPa) with depth increment under different scenarios. (Same lower case letters are not significantly different at P < 0.05 according to Duncan Multiple Range Test for separation of mean). Sc1- conventional rice-wheat system, Sc2- partial CA-based rice-wheat-mungbean system, Sc3- CA-based rice-wheat-mungbean system, Sc 4- CA-based maize-wheat-mungbean system.

Tillage and residue management significantly (P = 0.05) influenced SPR, which showed an increasing trend with depth to 25 cm and then decreased at the lower depths under all the scenarios (Figure 2). At 0–10 cm soil depth, highest value of SPR was recorded in Sc1 (1.35 MPa) at 5–10 cm, and the lowest in Sc2 (0.46 MPa) at 0–5 cm depth. No significant effect of different scenarios was observed on SPR in 10 to 30 cm soil depths, although below that it decreased with increasing depth in all the scenarios. SPR at 30–35 cm soil layer was significantly (P = 0.05) lower in Sc3 compared to Sc1 and Sc2 whereas Sc1 recorded significantly higher SPR values compared to the others beyond 35 cm depth. However, the SPR values irrespective of scenarios at 0–20 cm layers were below the critical value of 2-3MPa for optimum growth of wheat roots (Bengough and Mullins 1990). In general, SPR was higher under Sc1 compared to CA-based practices. Our results corroborated the findings of Blanco-Canqui et al. (2005) who also reported lower SPR under ZT- based management practices.

### Infiltration

**Figure 3.**
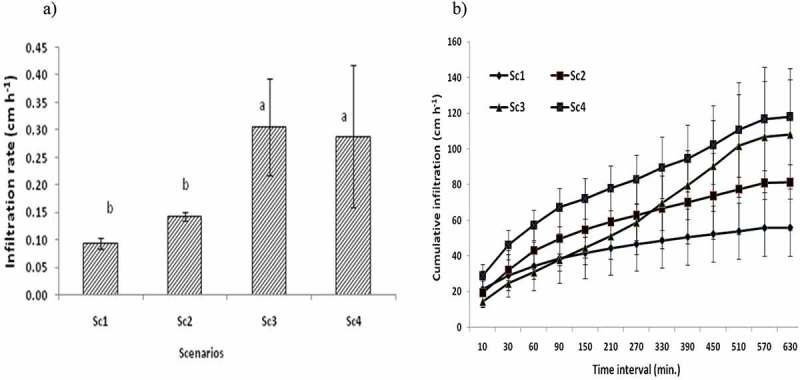
(a) Changes in infiltration rate and (b) cumulative infiltration at 0–15 cm soil depth under different scenarios. (Same lower case letters are not significantly different at P < 0.05 according to Duncan Multiple Range Test for separation of mean. Vertical bars indicate ± S.E. of mean of the observed values.) Sc1- conventional rice-wheat system, Sc2- partial CA-based rice-wheat-mungbean system, Sc3- CA-based rice-wheat-mungbean system, Sc 4- CA-based maize-wheat-mungbean system.

CA-based agricultural practices significantly influenced infiltration rate and cumulative infiltration. Scenario3 (0.31 cm h^−1^) and Sc4 (0.29 cm h^−1^) recorded highest infiltration rate whereas lowest was observed under Sc1 (0.09 cm h^−1^) (Figure 3(a)). Cumulative infiltration was increased with time interval. Sc4 showed highest cumulative infiltration in all the time intervals than others (Figure 3(b)). Cumulative infiltration in soils under Sc1, Sc2 and Sc4 increased with time except Sc3 where initially upto 60 min lowest cumulative infiltration was observed. After that suddenly it increased significantly and surpassed Sc1 and Sc2 at 90 min and 300 min, respectively (Figure 3(b)). Higher infiltration in ZT with residue retention might be due to direct and indirect influences of residue cover on water infiltration (Verhulst et al. 2010). Stable aggregates are formed under ZT with residue retention as compared to conventional tillage resulting in lesser breakdown of aggregates and less chances of surface crust formation. McGarry et al. (2000) reported higher infiltration rate and cumulative infiltration under ZT with residue retention as compared to conventional tillage. ZT with residue retention might have facilitated formation of continuous soil pores from the soil surface to depth which is attributed to the higher infiltration under CA-based systems (Verhulst et al. 2010; Gathala et al. 2011).

### Soil pH and electrical conductivity

Soil pH under Sc4 was significantly (P = 0.05) lower by 0.46 units compared to Sc1 at 0–15 cm depth (Table 1). However, Sc2 and Sc3 had soil pH similar to Sc1. In 15–30 cm layer, though soil pH showed lower values under Sc2, Sc3 and Sc4 but the differences were not significant. In general, soil pH increased with depth in all the scenarios. It has been reported earlier that the surface soil becomes more acidic under CA practices than that under conventional practice (Limousin and Tessier 2007; Singh et al. 2014). The lowering of pH in surface layer under CA-based scenarios has been attributed to build up of soil organic matter and release of organic acids upon decomposition in the surface layer (Singh et al. 2014). The EC values in both the soil depths were quite lower than the critical level of 4 dS m^−1^ in all the scenarios suggesting less chances of salt toxicity in plants on this soil (Table 1).

### Soil organic carbon and total nitrogen

The major effect produced by the CA-based scenarios was the higher accumulation of organic carbon (OC) and total N at the soil surface compared to Sc1 (Table 1). Highest OC was observed under Sc4 (7.7 g kg^−1^) followed by Sc3 (7.5 g kg^−1^). Conventional farmers practice (Sc1) showed lowest OC (4.5 g kg^−1^) at 0–15 cm soil depth. Highest OC (4.9 g kg^−1^) at 15–30 cm soil depth was observed under Sc2 compared to others. Compared to other scenarios, OC was 23–27% higher in Sc 2 at 15–30 cm depth. Total N contents under different scenarios followed the same trend of OC (Table 2). Total N concentration in 0–15 cm soil depth under Sc2 and CA-based scenarios (Sc3 and Sc4) was 29% to 36% higher (P = 0.05) than Sc1. At 15–30 cm depth, total N concentration was significantly higher in Sc2 compared to other scenarios. The increase in organic carbon content in surface soil under CA is in accordance with other studies (Lopez-Fando and Pardo 2009; Malecka et al. 2012). After 5 years of no tillage in grey pea-barley cropping system, SOC and N concentration was increased by 2.0 and 0.5 Mg ha^−1^, respectively at 0–5 cm soil depth in semi-arid Spain (Lopez-Fando and Pardo 2009). Malecka et al. (2012) reported highest SOC (10.2 g kg^−1^) and total N (1.12 g kg^−1^) at 0–5 cm soil depth after 7 years of no tillage in Poland. Higher quantity of residue additions (both above as well as below ground) and their slow decomposition due to less soil disturbance might have caused higher OC and total N concentrations in the surface layer under Sc3 and Sc4 (Du et al. 2010; Dikgwatlhe et al. 2014). The incorporation of crop residues by wet tillage in rice under Sc 2 may have resulted in higher OC and N contents compared to other scenarios in lower depths (15–30 cm soil depth).

**Table 2.** Effect of different CA-based scenarios on total N, available N, P, K and S contents in different soil depths after rice, 2013.

	Total N (%)		N (kg ha^−1^)		P (kg ha^−1^)		K (kg ha^−1^)		S (mg kg^−1^)	
Scenarios^a^	Soil depth (cm)									
	0–15	15–30	0–15	15–30	0–15	15–30	0–15	15–30	0–15	15–30
1	0.14 ± 0.02b	0.06 ± 0.01b	117 ± 1.52d	134 ± 1.70a	15.7 ± 0.70c	13.8 ± 1.53a	183.4 ± 7.0c	206 ± 2.1c	20.8 ± 0.84a	18.6 ± 0.88a
2	0.18 ± 0.01a	0.09 ± 0.01a	132 ± 0.96c	138 ± 2.75a	17.9 ± 0.46b	10.6 ± 1.12b	217 ± 4.0b	176 ± 2.7d	22.0 ± 0.13a	19.3 ± 0.55a
3	0.19 ± 0.01a	0.07 ± 0.01b	156 ± 2.29b	131 ± 1.22a	21.6 ± 0.53a	11.3 ± 0.65b	236 ± 2.7b	223 ± 3.1a	19.1 ± 0.86b	9.90 ± 0.55b
4	0.19 ± 0.01a	0.07 ± 0.01b	197 ± 2.40a	109 ± 1.64b	19.6 ± 1.94a	10.2 ± 0.10b	318 ± 3.0a	216 ± 2.2b	18.6 ± 0.74b	9.01 ± 0.41b

^a^Sc 1- conventional rice-wheat system, Sc 2- partial CA-based rice-wheat-mungbean system, Sc 3- CA-based rice-wheat-mungbean system, Sc 4- CA-based maize-wheat-mungbean system. For all variables n = 3 ± standard deviation. Values within the same column differ significantly at P = 0.05 when not followed by the same small letter (s) according to Duncan Multiple Range Test for separation of mean.

### Available macronutrients

The availability of N, P, K and S was significantly influenced by different CA based practices at both the soil depths (Table 2). At surface soil (0–15 cm), available N was 33% and 68% higher under Sc3 and Sc4 compared to Sc1, respectively. Available N concentration decreased with increase in soil depth, the decrease was significant under Sc4 (74%) and Sc3 (19.5%) while it remained similar under Sc1 and Sc2 compared to surface layer (Table 2). In Sc3 and Sc4, nutrients accumulated in the surface layer due to retention of higher amounts of residue rich in plant nutrients and minimal soil disturbance, whereas under conventional practices residues were removed and remaining stubbles thoroughly incorporated in the plough layer (0–20 cm) by tillage operations. CA-based practices, particularly conservation tillage are reported to cause greater accumulation of nutrients in surface layer compared to conventional tillage (Holanda et al. 1998; Lopez-Fando and Pardo 2009). The concentration of available N in soil under all the scenarios was quite lower than the lower limit of 260 kg N ha^−1^ which can be attributed to immobilization of inorganic N in soil (Locke and Hons 1988).

Available P concentration in soil decreased with soil depth. At 0–15 cm depth, Olsen P was 25% and 38% higher under Sc4 and Sc3 compared to Sc1 (Table 2), respectively, which might be attributed to higher residue retention which moderate the soil moisture and temperature congenial to crop growth at the soil surface. The trend was however, reversed at 15–30 cm depth; Sc1 recorded 22–35% higher available P compared to the other scenarios. Our results are in agreement with those reported by Murillo et al. (2004). Puddling in rice in Sc2 increased the P availability because of reduced soil conditions which increased the solubility of Fe, Al and Ca bound phosphates.

Like N and P, soil under CA-based scenarios (Sc3 and Sc4) had significantly higher available K concentrations compared to Sc1 at both the soil depths (Table 2). At 0–15 cm depth, Sc2, Sc3 and Sc4 had 19%, 29% and 74% higher available K concentration compared to Sc1, respectively (Table 2). At 15–30 cm depth, CA based scenarios (Sc3 and Sc4) recorded 6.5% and 18% higher concentrations of available K compared to Sc1 and Sc2, respectively. The higher available K concentration under Sc3 and Sc4 in both the soil depths might be attributed to additions of large amount of K through crop residues. Crop residues contain high concentrations of total K which is readily converted to available K in soil (IRRI 1984). Our results are in agreement with the findings of Murillo et al. (2004) and Malecka et al. (2012). The higher uptake of K (because of higher yield, data not shown) in Sc2 might have resulted in depletion of available K in the sub-surface layer of soil.

Available S concentration showed opposite trend with significantly higher values (P = 0.05) of 9–11% under Sc1 and 15–18% under Sc2 than that under Sc3 and Sc4 at 0–15 cm soil depth (Table 2). Similar trend was observed at lower soil depth also. Available S concentration decreased with depth. Lower S concentration in CA based systems might be due to the immobilization of sulphur by microbes (Alexander 1961).

### Available micronutrients

DTPA-extractable available micronutrient cations (Fe, Mn and Zn) were significantly influenced by different CA-based practices in the surface soil depth (Table 3). At 0–15 cm soil depth, available Zn concentrations were 51%, 57% and 93% higher under Sc4, Sc2 and Sc3 compared to Sc1, respectively. Available Zn at surface soils under Sc2 and Sc4 was statistically at par. The higher available Zn concentration at 0–15 cm soil depth in CA based scenarios (Sc2, Sc3 and Sc4) might be attributed to greater addition of Zn through crop residues and its accumulation in the surface layer (Lopez-Fando and Pardo 2009). In sub-surface layer, concentrations of available Zn were similar under all the scenarios. The concentration of all the micronutrient cations decreased markedly with soil depth irrespective of scenarios. The available Cu concentration was similar under different scenarios at both the soil depths. Concentration of available Fe was significantly higher under Sc2 compared to that under Sc1, Sc3 and Sc4 with the lowest values recorded under Sc4 (Table 3). The increase in concentrations of available Fe under Sc2 was 10%, 13% and 69% compared to Sc3, Sc1 and Sc4, respectively. A similar trend in available Fe was observed in 15–30 cm soil depth; Fe concentration was 38%, 50% and 55% higher under Sc2 compared to Sc1, Sc3 and Sc4, respectively. Higher concentrations of available Fe in Sc1 and Sc2 might be due to the greater availability of iron under reduced conditions in puddled transplanted rice which helps in conversion of less available Fe^3+^ fractions to easily available Fe^2+^ fractions (Ponnamperuma 1972). Like Zn and Fe, concentration of available Mn in soil was significantly influenced by different scenarios (Table 3). The highest concentration of Mn was recorded under Sc3 followed by Sc4 and the lowest concentration was observed under Sc2 at 0–15 cm depth. The Mn concentration under Sc3 was 13%, 21% and 32% higher compared to that under Sc4, Sc1 and Sc2, respectively. The available Mn concentrations at 15–30 cm soil depth were, however, similar under all the four scenarios (Table 3).

**Table 3.** DTPA extractable micronutrient cations under different conservation agriculture scenarios after harvesting of rice, 2013.

	Zn (mg kg^−1^)		Cu (mg kg^−1^)		Fe (mg kg^−1^)		Mn (mg kg^−1^)	
Scenariosa	Soil depth (cm)							
	0–15	15–30	0–15	15–30	0–15	15–30	0–15	15–30
1	4.75 ± 0.13c	0.66 ± 0.40a	2.70 ± 0.18ab	0.52 ± 0.30a	132 ± 1.24c	6.84 ± 1.44b	81.3 ± 1.45c	5.72 ± 0.87a
2	7.45 ± 0.33b	0.76 ± 0.21a	3.01 ± 0.15a	0.74 ± 0.29a	149 ± 1.65a	9.46 ± 0.99a	74.5 ± 0.73d	6.21 ± 1.21a
3	9.15 ± 0.07a	0.55 ± 0.19a	2.70 ± 0.25ab	0.55 ± 0.24a	136 ± 1.04b	6.31 ± 1.32b	98.6 ± 1.79a	6.82 ± 1.29a
4	7.17 ± 0.08b	0.50 ± 0.20a	2.55 ± 0.25a	0.50 ± 0.33a	87.6 ± 0.88d	6.09 ± 1.78b	87.3 ± 0.99b	6.11 ± 0.91a

^a^Sc 1- conventional rice-wheat system, Sc 2- partial CA-based rice-wheat-mungbean system, Sc 3- CA-based rice-wheat-mungbean system, Sc 4- CA-based maize-wheat-mungbean system. For all variables n = 3 ± standard deviation. Values within the same column differ significantly at P = 0.05 when not followed by the same small letter (s) according to Duncan Multiple Range Test for separation of mean.

### Correlation matrix

Significant correlations (P ≤ 0.01 and P ≤ 0.05) were observed among most of the soil properties, irrespective of soil depth. Soil BD was significantly and negatively correlated with all the soil properties except volumetric water content. OC had significant positive correlation with all the soil physical and chemical properties studied (Table 4). All the macro and micronutrients were significantly positively correlated with each other as reported by Mahashabde and Patel (2012). Our results corroborated with the findings of Kumar et al. (2014) who have reported significant positive correlation between OC and available S. A significant positive correlation (r = 0.96**) between total N and OC was reported by Nweke and Nnabude (2014) in tropical agro-ecosystems. Significant positive correlations between available S, total N and OC were also reported by Singh et al. (2009). Organic carbon in soil improved physical and chemical properties of soil under CA-based agricultural practices which is reiterated by the significant positive correlation between OC and other soil properties.

**Table 4.** Correlation matrix of different soil parameters irrespective of depth and scenarios.

	pH	EC	BD	VWC	OC	TN	Av N	Av P	Av K	Av S	Zn	Cu	Mn	Fe
pH	1													
EC	0.16													
BD	−0.08	−0.17												
VWC	0.01	0.31	0.13											
OC	−0.21	0.24	−0.50*	−0.08										
TN	−0.10	0.02	−0.67**	−0.37	0.74**									
Av N	0.30	0.40	−0.41*	0.06	0.63**	0.61**								
Av P	−0.04	−0.01	−0.47*	−0.36	0.72**	0.86**	0.69**							
Av K	−.65**	0.06	0.09	−0.03	0.52**	0.38	0.39	0.51*						
Av S	0.39	0.18	−0.66**	−0.31	0.42*	0.62**	0.63**	0.54**	−0.16					
Zn	0.01	−0.07	−0.64**	−0.50*	0.69**	0.95**	0.53**	0.87**	0.25	0.65**				
Cu	0.02	−0.12	−0.68**	−0.54**	0.59**	0.92**	0.48*	0.79**	0.18	0.68**	0.97**			
Mn	−0.06	−0.06	−0.58**	−0.52**	0.70**	0.95**	0.54**	0.89**	0.34	0.61**	0.99**	0.95**		
Fe	0.13	−0.14	−0.65**	−0.58**	0.55**	0.89**	0.40	0.76**	0.08	0.67**	0.97**	0.97**	0.94**	1

** – significant at the 0.01 level (2-tailed); * – significant at the 0.05 level (2-tailed); EC: electrical conductivity; BD: bulk density; VWC: volumetric water content; OC: oxidizable organic carbon; TN: total nitrogen; Av N: available nitrogen; Av P: available phosphorus; Av K: available potassium; Av S: available sulphur; Zn: DTPA-Zn; Cu: DTPA-Cu; Mn: DTPA-Mn; Fe: DTPA- Fe.

### Response of wheat to applied N and K under different scenarios

In the nutrient omission trial, wheat grain yield ranged between 3.83 to 5.48 t ha^−1^ across the scenarios (Table 5). Nitrogen response was observed up to 100% of the recommended N (160 kg ha^−1^) in Sc1, while in other scenarios it was observed up to 85% of the applied N (136 kg ha^−1^). The grain yield at the 100% N rate was 5.33 t ha^1^in Sc1 and it was similar to 70% of applied N in other respective scenarios (Sc2-5.37, Sc3-5.32 and Sc4-5.16 t ha^−1^). Partial and full CA-based scenarios saved 30% (48 kg N ha^−1^) N compared to farmer’s practice after 4 years of continuous cultivation with same management practices. The saving of fertilizer N was attributed to improvement of soil properties and nitrogen availability due to higher solubility of nutrients through moderation in soil moisture and temperature using crop residues retention under zero till conditions. Similarly, application of 50% of recommended K (60 kg K ha^−1^) produced the similar yields as 100% K in CA-based scenarios. In Sc1 and Sc2, application of 50% K recorded the lower yield compared to 100% K. At 0% K, Sc4 recorded the similar yields (5.05 t ha^−1^) to 100% K in Sc1 (5.05 t ha^−1^) and 50% K in Sc3 (5.06 t ha^−1^) and Sc2 (5.05 t ha^−1^). CA-based scenarios produced wheat grain yield similar to that with 100% K ha^−1^ while saving of 30 kg K ha^−1^ of potassic fertilizers which are being imported requiring foreign exchange of the Government of India.

**Table 5.** Yield of wheat (t ha^−1^) in N and K omission plots under different scenarios.

Treatment	Sc1	Sc2	Sc3	Sc4
N (% of 160 kg N ha^−1^)				
100	5.33 ± 0.8a	5.23 ± 0.5b	4.99 ± 0.5bc	5.30 ± 0.16ab
85	5.12 ± 0.07a	5.63 ± 0.17a	5.48 ± 0.11a	5.42 ± 0.04a
70	4.63 ± 0.06b	5.37 ± 0.03ab	5.32 ± 0.18ab	5.16 ± 0.12b
55	3.56 ± 0.06c	4.92 ± 0.09c	4.62 ± 0.06c	4.98 ± 0.14c
0	2.41 ± 0.02d	3.89 ± 0.06d	3.83 ± 0.10d	3.68 ± 0.10d
K (% of 60 kg K ha^−1^)				
100	5.00 ± 0.05a	5.25 ± 0.10a	5.01 ± 0.05a	5.35 ± 0.07a
50	4.52 ± 0.04b	5.10 ± 0.05b	5.06 ± 0.02a	5.40 ± 0.05a
0	4.36 ± 0.04c	4.46 ± 0.02c	4.50 ± 0.05b	5.05 ± 0.10b

^a^Sc 1- conventional rice-wheat system, Sc 2- partial CA-based rice-wheat-mungbean system, Sc 3- CA-based rice-wheat-mungbean system, Sc 4- CA-based maize-wheat-mungbean system. For all variables n = 3 ± standard deviation. Values within the same column differ significantly at P = 0.05 when not followed by the same small letter (s) according to Duncan Multiple Range Test for separation of mean.

## Conclusions

Results from the present study suggest that CA based cropping systems improved soil properties and availability of nutrients (N, P, K, Zn, Fe and Mn) in surface soil layer compared to conventional farmer’s practice. Appreciable amount of N and K fertilizers to the tune of 30% and 50% can be saved under CA-based management system after 4 years of continuous cultivation as revealed through nutrient omission study. By following CA based practices, we can save precious nutrient resources through building soil quality along with the well-established advantage of higher productivity (yield, water and energy) and profitability in North-West India.
